# UPPER LIMB MUSCLE STRENGTH AND WHEELCHAIR-RELATED ABILITIES FOLLOWING AN EXOSKELETON-ASSISTED WALKING PROGRAMME IN INDIVIDUALS WITH CHRONIC SPINAL CORD INJURY: AN EXPLORATORY STUDY

**DOI:** 10.2340/jrm.v56.19461

**Published:** 2024-11-21

**Authors:** Alec BASS, Mylène AUBERTIN-LEHEUDRE, Claude VINCENT, Cyril DUCLOS, Dany H. GAGNON, the MEWP group

**Affiliations:** 1 School of Rehabilitation, Faculty of Medicine, Université de Montréal, Montréal, QC; 2 Centre for Interdisciplinary Research in Rehabilitation of Greater Montreal (CRIR) – Centre Intégré Universitaire de Santé et Services Sociaux (CIUSSS) du Centre-Sud-de-l'Île-de-Montréal, Montréal, QC; 3 Department of Exercise Science, Université du Québec à Montréal, Montréal, QC; 4 Department of Rehabilitation, Faculty of Medicine, Université Laval, Québec, QC, Canada

**Keywords:** dynamometer, exoskeleton device, locomotion, muscle strength, rehabilitation, spinal cord injury, upper limb, wheelchairs

## Abstract

**Objectives:**

To measure the potential effects of an overground exoskeleton-assisted walking programme on upper limb strength and mass, as well as on wheelchair propulsion performances and abilities in individuals with chronic spinal cord injury.

**Design:**

Prospective, single-group, pre–post intervention study.

**Participants:**

Ten individuals with chronic (≥ 18 months) spinal cord injury who use a wheelchair as their primary mode of locomotion and who had little-to-no motor function in the lower limbs.

**Methods:**

Individuals completed a progressive 16-week exoskeleton-assisted walking programme (34 × 1-h sessions, 1–3 sessions/week). Upper limb muscle strength was measured with dynamometers (isokinetic, Jamar). Upper limb lean mass (dual-energy X-ray absorptiometry) was used to calculate relative strength. Field tests (20-m wheelchair propulsion, and slalom test) and the Wheelchair Skills Test Questionnaire determined performances and abilities. Wilcoxon signed-rank tests were used with the following criteria: *p* < 0.1, effect size ≥ 0.5, and relative variation > 5%.

**Results:**

Only natural velocity during the 20-m wheelchair propulsion test (i.e., fundamental wheelchair ability) changed following the intervention (*p* = 0.01, effect size = 0.82, relative variation = +14.5%).

**Conclusion:**

Overall, upper limb muscle function did not significantly and meaningfully change following the exoskeleton-assisted walking programme in this population. Additional research is needed to verify how changes in training volume would affect strength and advanced wheelchair-related abilities and performance, as well as the response in individuals who are deconditioned or novices to wheelchair use (e.g., subacute spinal cord injury).

## LAY ABSTRACT

Increased muscular demand and weight-bearing on the upper limbs elevate the risk of shoulder impairments in long-term manual wheelchair users with spinal cord injury. As such, upper limb exercise training is recommended. Exoskeleton-assisted walking may be a useful modality as it solicits key upper limb muscles. This study aimed to measure the potential effects of an overground exoskeleton-assisted walking programme on upper limb strength and mass, as well as on wheelchair propulsion performances and abilities in individuals with spinal cord injury. Ten participants with chronic spinal cord injury, who had little-to-no motor function in the lower limbs, completed a progressive 16-week exoskeleton-assisted walking programme (34 × 1-h sessions, 1–3 sessions/week). Only natural velocity during the 20-m wheelchair propulsion test increased after training. These findings confirm that the proposed training volume may not increase muscle function in long-term manual wheelchair users with spinal cord injury. Additional research is needed in individuals who are deconditioned or novices to wheelchair use (subacute spinal cord injury).

For nearly 60% of individuals living with a spinal cord injury (SCI), their upper limbs become the main drivers of mobility and are crucial for maximizing independence in daily activities (1). The performance of these activities is possible given the multiaxial mobility resulting from several versatile joints and the force-generating capacity of large thoraco-humeral muscles (2–5). This provides mobility and stability at the trunk and upper limbs. However, the chronic increase in upper limb solicitation and the increased risk exposure due to repetitive, intense, and rapid loading during wheelchair propulsion and other wheelchair-related activities leads to a high incidence of overuse injuries and pain in this population, particularly at the shoulders (6–10). The consequences of such impairments are wide reaching, affecting wheelchair propulsion and the performance of many wheelchair-related activities, which in turn jeopardize independence and safety in other activities of daily living. This vicious circle ultimately reduces participation in occupational, social, and leisure activities, and reduces quality of life (9).

Preventing secondary upper limb injury is therefore a priority in this population. To achieve this goal, regular exercise is recommended to improve muscle strength and function (6, 7, 10–13). Indeed, improving the strength of upper limb muscles reduces relative muscular demand when performing activities of daily living (e.g., wheelchair propulsion, transfers, etc.) and is associated with improved overall wheelchair function (14, 15). This helps to alleviate the risk of overuse injuries as shown in individuals without chronic disabilities (16). For individuals living with SCI, it is recommended that moderate to vigorous resistance exercises, targeting functioning muscles, are performed at least twice a week (3 sets of each exercise) (13). In addition, 30 min of moderate to vigorous intensity aerobic exercise, performed 3 times per week, is recommended (13).

Rehabilitation professionals, and individuals alike, must conciliate key training parameters, ensuring that prescribed exercises meet guidelines (especially in terms of frequency and intensity), while also avoiding injury by overworking muscle groups and joints that are already highly solicited. This becomes challenging, especially as a commonly used modality to train upper limbs and cardiorespiratory fitness is the arm crank ergometer (which is known to solicit a repetitive cyclic movement pattern somewhat comparable to wheelchair propulsion). As such, exoskeleton-assisted walking provides a unique and new opportunity to train the upper limbs, while also leading to potential cardiorespiratory and lower limb musculoskeletal health benefits (17–19). More specifically, overground exoskeleton-assisted walking activates the trunk, shoulder, and arm muscles to various extents to: (*i*) maintain standing balance, (*ii*) control anterolateral body weight shifting required to trigger stepping, and (*iii*) reduce lower limb loading to ensure a soft heel strike at each step (17, 20). Indeed, muscle recruitment analysis of exoskeleton-assisted walking in individuals without lower limb motor function has shown normalized muscle activation ratios at the trunk reaching 75–150% of maximal voluntary contraction measured in supine or prone positions (17). This highlights a high degree of muscle activation in this population, possibly facilitated through intact vestibulospinal pathways activated through walking (and not in quiet supine, prone, or seating positions) (17). Moreover, a recent detailed biomechanical analysis of exoskeleton-assisted walking concluded that the upper limb joint moments (shoulders, elbows) were greater in individuals with complete spinal cord injury than in individuals with incomplete spinal cord injury (20). Although joint forces in the shoulders were significant, they were lower than those previously reported during pivot transfers (20). Overall, these results imply significant upper limb and trunk recruitment during walking in individuals with little-to-no motor function in the lower limbs, while remaining less taxing on the shoulder joints than certain activities of daily living (i.e., transfers). Importantly, although exoskeleton-assisted walking solicits similar musculature to wheelchair propulsion, movement patterns and force orientation are different (17, 20). Thus, an exoskeleton-assisted intervention may mitigate the risk of shoulder pain and secondary impairments associated with training, while still contributing to improving muscle strength and function.

The current paper aimed to measure the potential effects of an overground exoskeleton-assisted walking programme on upper limb muscle strength and mass, as well as on wheelchair performance and abilities, in individuals with chronic SCI. It was hypothesized that the walking programme would lead to significant increases in upper limb muscle strength and mass, which in turn would translate into the improvement of wheelchair performances and abilities.

## MATERIAL AND METHODS

### Study design and participants

This study was approved by the ethics committee of the *Centre de recherche interdisciplinaire en réadaptation du Montréal métropolitain* (CRIR-1338-0518) on 14 March 2019. The protocol has been published previously and was registered with the U.S. National Library of Medicine (clinicaltrials.gov) on 7 June 2019 (registration number NCT03989752) (21). This prospective pre–post intervention study included a convenience sample of adults (≥ 18 years old) with chronic (i.e., ≥ 18 months) complete or incomplete SCI. Sample size calculated *a priori* was 20 participants, based on variability of absolute change in body composition and bone mineral density measured in a pilot study in our laboratory and is described in greater detail in the published protocol (21). Individuals were contacted through a database of previous laboratory participants (who accepted to be contacted again), or reached out directly to the research team through word of mouth. To be included, individuals needed to: (*i*) use a wheelchair as their primary mode of locomotion; (*ii*) understand French or English and; (*iii*) reside (or be able to arrange to reside) within 75 km of the research site. Individuals were excluded if they had: (*i*) neurological impairments unrelated to the SCI (e.g., multiple sclerosis); (*ii*) a concomitant or secondary musculoskeletal impairment limiting their ability to ambulate safely (e.g., hip heterotopic ossification); (*iii*) a history of fragility fractures within the past year, or any other condition that may preclude safe lower limb weight-bearing, walking, or exercise tolerance (e.g., unstable cardiovascular or autonomic system, renal insufficiency, etc.). Individuals also had to meet criteria specific to the wearable robotic exoskeleton used in this study (Ekso GT, Ekso Bionics), including maximum anthropometric measures and minimal lower- and upper-limb range of motion. Inclusion and exclusion criteria are described in greater detail in the previously published (open access) protocol (21).

### Measurement times and intervention

Due to constraints imposed by the COVID-19 pandemic, the 4 measurement times in the published protocol (i.e., 1 month pre, immediate pre, immediate post, and 2 months post) were not possible. Only pre-intervention and immediate post-intervention assessments were possible. A participant’s pre-intervention measurements represented the average value between measurements taken 4 weeks before and immediately before the intervention. Averaging these measurements allowed us to account, to a certain extent, for natural variability related to repeated measures. Post-intervention measurements were solely taken within 7 days following the end of the intervention.

**Fig. 1:**
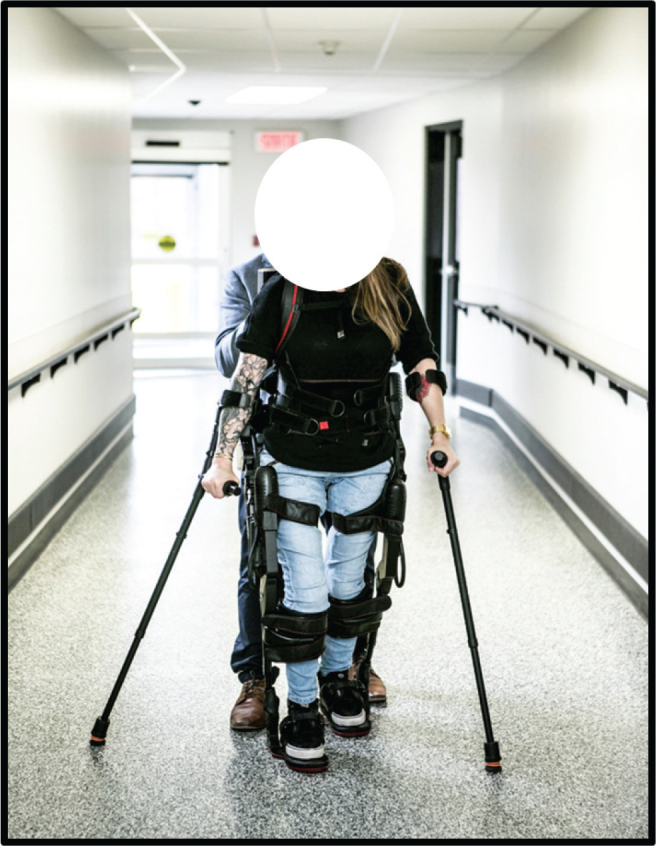
Person walking in the exoskeleton used in this study.

Following pre-measurements, individuals engaged in a wearable robotic exoskeleton-assisted overground walking programme consisting of 34 individual sessions (60 min/session) over a 16-week period. A published algorithm was used to adapt training volume and progression based on an osteoporotic profile determined by dual-energy X-ray absorptiometry (DEXA) (22). Individuals were classified as 1 of 3 profiles: osteoporosis, osteopenia, or preserved bone mineral density. The number of steps prescribed per training session was then modulated, starting at 300, 400, and 500, and progressed weekly by 10, 15, and 20%, respectively, according to their assigned profile. For all profiles, individuals began with 1 training session per week, and progressed to 3 training sessions per week by the end of the programme. The detailed training protocol has been published previously (21, 22). To maintain a moderate-to-vigorous exercise intensity during the sessions, walking speed, resting time, assistive devices (i.e., walker or crutches), and assistance provided by the therapist were modulated to ensure a rate of perceived exertion of ≥ 3/10. All training sessions were supervised by a certified physiotherapist, with the help of a second physiotherapist or a physiotherapy technician when necessary. Fig. 1 shows a person walking in the exoskeleton used in this study.

### Outcome measures

Sociodemographic data were collected prior to initiating the walking programme. Self-reported levels of weekly physical activity were also collected, and participants were instructed to maintain the same level of physical activity throughout the duration of the study. Overall, 5 indicators were measured to assess muscle and wheelchair function.

### Upper limb muscle

Because improvements in muscle strength are attributed to both neural adaptations and muscle hypertrophy, in addition to absolute muscle strength, lean mass (indication of muscle hypertrophy) and relative muscle strength (indication of neural adaptation) were also collected (23). For all measurements, the self-reported non-dominant arm was used.

### Absolute functional strength

**Fig. 2:**
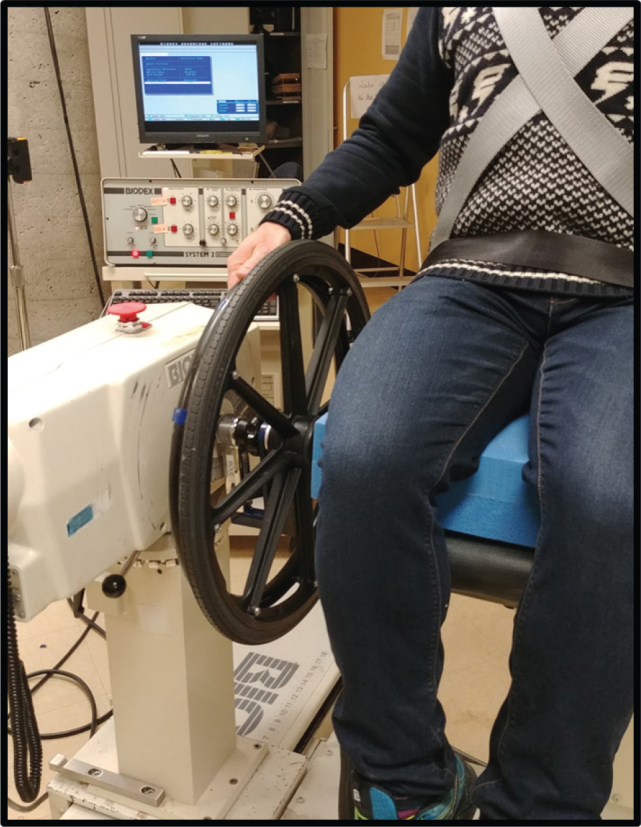
Wheelchair wheel adapted to become mountable onto the shaft of an isokinetic dynamometer system.

To obtain an activity-specific functional measure of absolute upper-limb functional strength, the axle of a wheelchair wheel was adapted to become mountable onto the shaft of an isokinetic dynamometer system (Biodex, see Fig. 2). Participants transferred onto the adjustable chair that was then positioned to duplicate their personalized wheelchair adjustments (i.e., chair height, seat angle etc.). Two shoulder straps crossed over the thorax, as well as an additional strap around the hips, were used to stabilize the participants in the chair. Chair positioning was noted to ensure consistency during repeated measures. The dynamometer was programmed to allow a total wheel excursion of 75° (24, 25). The highest vertical point of the handrim was used as the reference (i.e., 0°). Using this reference point, the dynamometer was programmed to allow 20° of backward rotation (i.e., –20°) and 55° of forward rotation (i.e., +55°) (24, 25).

*Isometric, isokinetic and isotonic push and pull tasks.* These tasks were measured in the order described hereafter for all participants. For the first set of tasks, participants completed three 5–10 s isometric pushes at 0°, separated by 1-min rest periods. This was followed by 3 x 5–10 s isometric pulls at +55°, also separated by 1-min rest periods. The mean peak forces for the 3 isometric pushes, as well as the 3 isometric pulls, were calculated and used as the outcome measures. For the second set of tasks, participants completed three isokinetic pushes (30°/s) preceded by 2 s of preloading, each separated by 1-min rest periods. This was followed by 3 isokinetic pulls with the same parameters. All isokinetic tasks were performed throughout the entire available wheel excursion (i.e., between –20° and +55°). The mean peak forces for the 3 isokinetic pushes, as well as the 3 isokinetic pulls, were calculated and used as the outcome measures. For the third set of tasks, participants completed 5 consecutive isotonic (resistance = 14 Nm) push-and-pulls (i.e., 1 push followed immediately by 1 pull and so on) as fast as possible. No rest periods were allowed between trials for the isotonic tasks. All isotonic tasks were performed throughout the entire available wheel excursion (i.e., from –20° to +55° and from +55° to –20°). The mean peak velocities for the best 3 isotonic pushes, as well as the best 3 isotonic pulls, were calculated and used as the outcome measures. Standardized verbal encouragements were provided during all trials of all tasks. For all of these outcomes, brute data were exported from the laboratory’s software (LabVIEW) and imported into Excel (Microsoft 365; Microsoft Corp, Redmond, WA, USA). Time–force (or time–velocity) curves were re-created and visually inspected (AB) for artefacts. Peak force or velocity values, for each trial, were extracted and averaged directly in Excel.

*Grip strength.* While seated in their wheelchair, participants held a dynamometer (Jamar Plus, Sammons Preston, Performance Health, Sutton in Ashfield, UK) in neutral pro-supination position with the elbow flexed at 90° and firmly pressed against the thorax. Participants completed 3 x 5–10 s maximal isometric grips, each separated by 1-min rest periods. The mean peak forces for the 3 tests were calculated and used as the outcome measures. Standardized verbal encouragements were provided. This measure was used as a good indicator of overall upper limb muscle strength and function (26).

### Lean mass

Full-body DEXA scans were performed on a separate day, in the morning, following an 8-h fast. Participants were asked to empty their bladder if they had not done so within the hour. Scans were taken following the standardized protocol recommended by the manufacturer of the DEXA (General Electric Lunar Prodigy; standard mode; software version 12.30.008; GE Healthcare, Chicago, IL, USA). Calibration was executed daily with a standard phantom prior to each test. Participants lay supine, free of jewellery or any other metallic objects. Clothing worn was noted and participants were asked to wear the same clothing for repeated scans. Quantitative analysis was provided automatically by the machine’s software. Lean mass (kg) of the non-dominant arm was selected as the outcome measure.

### Relative muscle strength

Relative muscle strength was calculated by dividing the measured forces (i.e., isometric and grip strength) by unilateral arm lean mass (i.e., DEXA). Relative muscle strengths are expressed in Newton-metres per kilogram of mass (Nm/kg) or in kilograms of force per kilogram of mass (kg/kg).

### Wheelchair performances and abilities

*Wheelchair performance tests*. Wheelchair abilities were assessed with the 20-m wheelchair propulsion test (at natural and maximal velocities) and the timed manual wheelchair slalom test (14, 27, 28). These tests were performed after a rest period (5–10 min) following the functional upper-limb strength testing (i.e., dynamometer tasks). All wheelchair performance tests were performed in the order described hereafter, separated by 3–5-min rest periods. No verbal encouragements were provided during the tests and participants were only informed of their performance after completion of all trials.

The 20-m wheelchair test was performed in a long, levelled, and unobstructed corridor. Start and finish lines were separated by 20 m and marked on the floor with bright-coloured tape and bright cones with flags (14, 28). The starting position was standardized: participants aligned their front caster wheels to the start line and placed their hands on their knees, palms facing down. Participants performed 3 trials at a self-selected natural velocity, followed by 3 trials at maximal velocity. A stopwatch began following a 3-s countdown and was stopped when the front caster wheels touched the finish line. Velocity was calculated (20 metres divided by seconds measured.) and means for natural and maximal velocity trials were used as the outcome measures.

The timed manual wheelchair slalom test was performed in the same corridor, along which a slalom trajectory was created using 7 bright-coloured plastic cones with flags, aligned in a straight line (14, 27, 28). Start and finish lines were separated by 18 m and marked on the floor with bright-coloured tape. Three metres separated the start line and the first cone, the first and second cones, as well as the second and third cones. Two metres separated the third and fourth, as well as the fourth and fifth cones. One metre separated the fifth and sixth, as well as the sixth and seventh cones. Finally, 3 m separated the seventh cone and the finish line. The same standardized starting position, described previously, was used. Participants were allotted 1, slow-paced practice run to familiarize themselves with the course and the distance between the cones. Participants then performed 3 trials at maximal velocity. A stopwatch began following a 3-s countdown and was stopped when the front caster wheels touched the finish line. Velocity was calculated (18 metres divided by seconds measured) and mean velocity was used as the outcome measure. In addition to providing a measure of wheelchair performance, this test served as a surrogate measure of functional trunk stability (14). Indeed, this test was designed to functionally assess asymmetrical force generation in the upper limbs and the trunk (27). In fact, measures of isometric upper limb muscle strength and dynamic postural stability (i.e., trunk muscles) have been shown to be strong predictors and key determinants of the performance on the slalom test (14).

*Wheelchair abilities.* Following the wheelchair tests, participants were given questionnaires, with the option of completing them on-site or taking them home to be returned within a week. The Wheelchair Skills Test Questionnaire (version 5.0) was used to assess wheelchair mobility and wheelchair-related skills. The questionnaire is composed of 34 questions that evaluate capacity (Can you do it? 0 = no, 3 = very well), confidence (How confident are you? 0 = not at all, 3 = very) and frequency (How often do you do it? 0 = never, 3 = always). Capacity, confidence, and frequency scores were calculated and expressed as percentage scores. Higher scores indicated greater wheelchair skills (29).

### Statistics

Descriptive statistics were used to characterize participants. As the sample size was limited and some outcome measures were not normally distributed, nonparametric tests (i.e., Wilcoxon signed-rank test) were used to compare pre vs post intervention measurement data. Standardized effect sizes (*r*) were calculated by dividing the z value by the square root of the number of observations and interpreted as being negligible (< 0.1), small (≥ 0.1), medium (≥ 0.3), or large (≥ 0.5) (30, 31). Relative pre vs post median variations (%) were also computed for all outcomes. Given the exploratory nature of this study, 3 criteria needed to be met to reach significance and meaningfulness: (*i*) the alpha for statistical tests needed to be < 0.10 to balance risk of false negatives due to an anticipated lack of statistical power, (*ii*) calculated effect sizes needed to be large (i.e., ≥ 0.5) for an outcome to be deemed clinically relevant, and (*iii*) relative variation needed to be greater than 5% to be considered as a change exceeding natural variability and potential measurement errors as used in previous work (32). All statistical analyses were conducted using SPSS version 28 (IBM Corp, Armonk, NY, USA).

## RESULTS

Characteristics of the participants are summarized in Table I. Due to restrictions imposed in response to the COVID-19 pandemic, a total of 10 participants (mean age 46 years; 4 females, 6 males) completed the programme. The sample was heterogeneous with regard to SCI neurological levels (C3 to T12), severity (American Spinal Injury Association Impairment Scale: A to C) and time to injury onset (3 to 18 years). Most participants (8/10) had full motor function of the upper limbs (i.e., upper extremity motor scores of 50/50) and 7 (7/10) met current SCI-specific guidelines for weekly physical activity volume (i.e., at least 90 min/week of aerobic exercises) (13). Regarding physical activity, 2 participants had very high weekly volumes. While 1 participant was a paralympic athlete, the other followed a strict regimen of arm ergometry 6 days per week. All individuals included in the study were right-hand dominant; therefore, all muscle outcomes were taken in the left arm.

**Table I:** Description of the participants (*n* = 10)

#	Sex	Age (years)	BMD profile	Walking programme progression	Neurological level (AIS)	UEMS (/50)	LEMS (/50)	Dominant hand	SCI duration (y)	Physical activity (min/week)	Weight (kg)	Height (m)	BMI (kg/m^2^)	Total body fat (%)
1	Male	41	Preserved	Fast	T8 (A)	50	0	Right	9.6	180	66.7	1.71	22.8*	34.1*
2	Male	36	Preserved	Fast	T6 (A)	50	0	Right	11.6	45	99.7	1.92	27.0*	39.5*
3	Male	67	Preserved	Fast	T10 (A)	50	0	Right	12.0	0	92.3	1.88	26.1*	37.8*
4	Male	60	Preserved	Fast	T11 (A)	50	0	Right	3.3	135	90.6	1.74	29.9*	38.7*
5	Female	35	Preserved	Fast	C3 (C)	25	0	Right	3.6	840	50.2	1.65	18.4	29.0
6	Male	32	Osteopenia	Moderate	T3 (A)	50	0	Right	8.6	30	73.5	1.75	24.0*	24.6*
7	Female	48	Osteopenia	Moderate	T12 (B)	50	5	Right	45.5	92.5	62.4	1.60	24.4*	51.8*
8	Female	42	Osteopenia	Moderate	T3 (A)	50	0	Right	7.7	360	70.7	1.66	25.7*	44.4*
9	Female	55	Osteoporosis	Slow	T4 (A)	50	0	Right	7.8	110	61.2	1.66	22.2*	43.0*
10	Male	47	Osteoporosis	Slow	C5 (A)	28	0	Right	18.3	105	81.3	1.86	23.5*	42.7*
Mean		46.3							12.8	189.8	74.9	1.70	24.4	38.5
SD		10.9							11.6	236.7	15.0	0.10	2.9	7.4

* Identifies obesity using criteria recommended by Paralyzed Veterans of America (BMI ≥ 22 kg/m^2^ or body fat > 22% in men and > 35% in women) (48). BMD profile = Pre-intervention bone mineral density profile of the left hip as measured by dual-energy X-ray absorptiometry (DEXA); AIS = American Spinal Injury Association (ASIA) Impairment Scale; UEMS = upper-extremity motor score on the American Spinal Injury Association (ASIA) Impairment Scale; LEMS = lower-extremity motor score on the American Spinal Injury Association (ASIA) Impairment Scale; SCI duration = spinal cord injury duration in years; Physical activity = self-reported number of minutes (min) of physical activity per week; Weight in kilograms (kg), Height in metres (m); BMI = body mass index in kilograms per square metre (kg/m^2^); Total body fat percentage as measured by dual-energy X-ray absorptiometry (DEXA); SD = standard deviation.

### Upper limb muscle

Outcome measures for muscle function are summarized in Table II. Of note, grip strength was unable to be measured for 2 participants as their SCI (C3 and C5) did not allow sufficient motor function for this task. Outcomes varied by approximately –8 to +14% following the intervention. A total of 3 outcomes (3/11) decreased, while 8 (8/11) increased. However, none of the outcome measures met all 3 criteria for significance and meaningfulness.

**Table II:** Summary of left arm musculature outcome measures (*n* = 10)

Outcomes	Pre-intervention	Post-intervention	*p*-value	Effect size	∆ (%)
Absolute functional strength					
Isometric pushing force (Nm)	62.7 (48.7, 77.7)	57.9 (47.7, 78.3)	0.88	0.05 (N)	–7.7
Isokinetic pushing force (Nm)	56.5 (42.7, 71.9)	59.6 (46.9, 75.6)	0.24	0.37 (M)	+5.4
Isotonic pushing velocity (°/sec)	281.4 (261.2, 289.7)	282.3 (258.3, 295.4)	0.17	0.44 (M)	+0.3
Isometric pulling force (Nm)	76.4 (58.7, 104.4)	87.3 (71.2, 109.1)	0.65	0.15 (S)	+14.2
Isokinetic pulling force (Nm)	73.2 (61.4, 104.5)	79.8 (66.4, 115.6)	0.65	0.15 (S)	+9.0
Isotonic pulling velocity (°/sec)	287.6 (265.6, 297.1)	290.3 (282.6, 304.3)	0.05*	0.63 (L)	+0.9
Grip strength (kg)^a^	39.8 (29.9, 44.7)	38.9 (30.8, 46.5)	0.33	0.35 (M)	–2.3
Arm lean mass (DEXA)					
Left arm lean mass (kg)	3.112 (2.265, 3.959)	3.289 (2.337, 3.707)	0.11	0.50 (M)	+2.1
Relative muscle strength					
Relative isometric pushing force (Nm/kg)	21.5 (18.9, 22.4)	21.3 (17.5, 25.0)	0.96	0.02 (N)	–0.9
Relative isometric pulling force (Nm/kg)	27.9 (17.7, 36.0)	28.9 (27.4, 32.2)	0.29	0.34 (M)	+3.6
Relative grip strength (kg/kg)^a^	12.6 (9.9, 13.2)	12.9 (11.2, 13.5)	0.26	0.40 (M)	+2.3

a *n* = 8. Data presented as median (1st quartile, 3rd quartile).

* Statistically significant difference (*p* ≤ 0.10) for Wilcoxon signed-rank tests. Standardized effect sizes interpreted as N = negligible (< 0.1), S = small (≥ 0.1), M = medium (≥ 0.3), or L = large (≥ 0.5), ∆ = relative variation (expressed as a percentage) between medians (positive indicates increase in value from pre to post measurement).

### Wheelchair performances and abilities

Outcome measures for wheelchair performances and abilities are summarized in Table III. Outcomes varied by approximately –7 to +15% following the intervention. A total of 4 outcomes (4/6) decreased, while 2 (2/6) increased. However, only natural velocity measured on the 20-m propulsion test met all 3 criteria with a *p* = 0.01, a large effect size (0.82), and a relative increase of 14.5% post-intervention.

**Table III:** Summary of wheelchair performances and abilities (*n* = 10)

Outcomes	Pre-intervention	Post-intervention	*p*-value	Effect size	∆ (%)
Wheelchair performance					
*** 20-m – Natural velocity (m/s)***	1.25 (1.16, 1.45)	1.43 (1.29, 1.46)	***0.01***	***0.82 (L)***	***+14.5***
20-m – Maximal velocity (m/s)	2.07 (1.75, 2.15)	1.99 (1.89, 2.10)	0.33	0.31 (M)	–3.7
Slalom – Maximal velocity (m/s)	1.13 (1.02, 1.19)	1.11 (1.07, 1.17)	0.33	0.31 (M)	–1.2
WSTQ					
Self-reported abilities (%)	72 (54, 91)	67 (62, 86)	0.44	0.24 (S)	–6.9
Self-reported confidence (%)	91 (80, 96)	86 (80, 95)	0.64	0.15 (S)	–5.0
Self-reported frequencies (%)	74 (61, 87)	77 (69, 84)	0.33	0.31 (M)	+4.8

Data presented as median (1st quartile, 3rd quartile). Bold italic indicates variables meeting following 3 criteria: statistically significant difference, effect size ≥ 0.5, relative median difference ≥ 5%.

*Statistically significant difference (*p* ≤ 0.10) for Wilcoxon signed-rank tests. Standardized effect sizes interpreted as N = negligible (< 0.1), S = small (≥ 0.1), M = medium (≥ 0.3), or L = large (≥ 0.5), ∆ = relative variation (expressed as a percentage) between medians (positive indicates increase in value from pre to post measurement). WSTQ = Wheelchair Skills Test-Questionnaire version 5.0 where higher scores indicate greater wheelchair skills.

## DISCUSSION

To our knowledge, this is the first study to investigate how upper limb muscle, as well as wheelchair propulsion performance and abilities, are affected immediately after the completion of a 16-week exoskeleton-assisted walking programme. Overall, in long-term manual wheelchair users with an SCI, this programme maintained upper limb muscle strength and wheelchair-related abilities (i.e., skills and performance), as only 1 outcome (i.e., natural wheelchair velocity) changed significantly and meaningfully.

### Exoskeleton-assisted walking programme did not alter upper limb muscle function

No statistically significant and meaningful changes in upper limb muscle strength outcomes, including the slalom test (i.e., surrogate measure of dynamic trunk stability), were detected. Only natural velocity during the 20-m wheelchair propulsion test, an outcome related to fundamental wheelchair performance and abilities, increased significantly and meaningfully after the exoskeleton-assisted walking programme. It should be noted that the natural velocity measured post-intervention in the present study is faster than that typically reported in this population, which has been previously reported to range between 1.2 and 1.3 m/s (28, 33). Due to the nature of this study, this may highlight a confirmation bias, as participants were not blinded to the intervention. Moreover, no other wheelchair outcomes changed following the intervention. Thus, the apparent effect on more advanced abilities may be limited. Additionally, although these results do not support our initial hypothesis, they do align with handicap creation conceptual models (absence of change in musculature translated to absence of change in advanced wheelchair performance and abilities). Nonetheless, although the training programme did not improve muscle strength or mass, it did not have negative effects on these outcomes. This result remains an important finding, as exoskeleton-assisted walking may represent a safe physical activity intervention for this population and may help to maintain or improve other physical (e.g., cardiometabolic) and mental health parameters (19, 34–37).

### Suboptimal training load and task specificity with the exoskeleton-assisted walking programme

Progressive overload (i.e., training progression) is an important factor to improve muscle strength and function (38). However, this parameter may not have been optimal in the present study because of the effects associated with learning the proper walking technique while in the exoskeleton and the slow loading progression imposed to mitigate lower limb fracture risk. Overground exoskeleton-assisted walking recruits thoracic and upper limb musculature as individuals must continuously generate forces in inferior and posterior directions, relative to their body. This is done using walking aids (i.e., walker, crutches) and is necessary to maintain balance and to transfer body-weight in an anterolateral direction during walking (17, 20). Importantly, assuming an individual’s weight remains the same, the forces needed to be generated would remain relatively stable throughout the training programme (i.e., no progression). Moreover, with learning of proper technique, walking efficiency improves (i.e., decrease in relative intensity of a set walking speed) and may reduce muscular demand for balance control and weight shifts (39). Although walking speed increased from week to week during the training programme, most individuals eventually attained a plateau as maximal walking speed is limited with the exoskeleton (40). Thus, adhering to the principle of training progression can be challenging and may not have been respected throughout the entire training programme, limiting potential muscular and performance improvements. This hypothesis requires further investigation, including, for instance, electromyographic analysis of muscular activity to determine variations in overall muscle activation and relative demands throughout the advancement of the walking programme.

Task specificity is another important factor for improving muscle function and performance (38). The overall nature of the walking programmw (i.e., sustained effort over a long duration) would most likely lead to improvements in muscle oxidative capacity (i.e., aerobic metabolism). This has the potential to improve natural wheelchair velocity, as it is a sustained low-to-moderate intensity effort during activities of daily living (41). However, effects on muscle strength and hypertrophy, as well as short bursts of maximal effort (such as during maximal wheelchair velocity and the slalom test), would likely be limited as these activities predominantly elicit anaerobic metabolic pathways. As such, including high-intensity exercise training in movements that are specific to wheelchair propulsion would be an important strategy. Adjunct exercises could encompass, for instance, resistance training and/or sprint-style wheelchair training (42-44). Moreover, advanced wheelchair abilities, such as being able to hold a wheelie or ascend or descend stairs in a wheelchair, are also likely to require specific training (especially as the relationship with exoskeleton-assisted walking remains limited) (45). Overall, training programmes encompassing a variety of exercises, each targeting specific characteristics, are expected be the most beneficial (46).

### Exoskeleton-assisted walking programme offered as reconditioning programme for potential health benefits

The results of the present study may have also been impacted by the fact that the participants were long-term wheelchair users with chronic SCI. As such, the participants could have been characterized as having “highly trained” upper limbs and mastering “advanced” wheelchair skills. This is further highlighted by comparing pre-intervention measurements with the literature. First, grip strength (i.e., 39.8 kg for the non-dominant left hand) surpassed reference values in the Canadian population (i.e., 37.7 kg for the maximal value between left and right hands), supporting the notion that participants’ upper limbs were already “highly trained” (47). Second, wheelchair performance (i.e., natural and maximal wheelchair velocities, slalom test) were all within or above the ranges expected in this population, indicating that individuals had already mastered the fundamentals of wheelchair propulsion and related skills (27, 28, 33). Third, individuals reported high levels of ability (72%), confidence (91%), and frequency (74%) on the Wheelchair Skills Test-Questionnaire, indicating that they had most likely already reached a high level of skill with their wheelchair. As such, combined with the previous suggestion that training parameters may have been suboptimal and not specific to advanced skills, the potential for improvement was likely limited within this sample. Whether similar results would be observed for users who are less confident, who use their wheelchair less frequently, or who report lower skill levels (i.e., in individuals with subacute SCI) remains to be determined.

### Study limitations

Interpretation of the results in the present study should be conducted with its limitations in mind. First, due to numerous challenges associated with the COVID-19 pandemic, recruitment of the initially planned sample size (i.e., 20) was not possible (21). Consequently, statistical power of the analyses is reduced. Moreover, the relatively small sample size in the present study meant that additional subgroup analyses were not possible (e.g., sex, bodyweight, obesity status, response to intervention). Second, the sample included in this study was heterogeneous with regard to SCI level and severity, although upper and lower limb motor function remained rather homogeneous, as is evidenced in Table I with the upper and lower extremity motor scores (UEMS and LEMS). Nevertheless, the degree of motor function at the trunk was not assessed and may have influenced the stability of the trunk during walking, which would in turn influence the level of muscle recruitment in the upper limbs. Third, by including only individuals with chronic SCI, the sample of individuals was inherently less inclined to benefit from the walking programme in terms of upper limb musculature and functional abilities, especially those related to wheelchair mobility. Fourth, the wheelchair wheel attachment used in this study has not undergone formal evaluation of its psychometric properties. Nevertheless, as this wheel was welded onto a pre-existing arm designed for the Biodex system, it is not expected to have significantly affected the performance of the dynamometer. Fifth, the present study focused exclusively on wheelchair performance and abilities (i.e., skills and performance). The absence of the assessment of other relevant functional activities, like the performance of transfers, may not have captured other positive effects of the intervention. Likewise, the addition of an interview to capture participants’ perspectives in terms of training effects on upper limb muscle strength or wheelchair ability would have provided valuable information for consideration.

### Clinical implications

This discussion has included several important clinical implications. First, exoskeleton-assisted training alone may not be sufficient to increase upper limb muscle strength in individuals with chronic SCI. Clinicians should consider specific exercises, such as resistance training, if their main goal is to increase muscle strength. At the very least, clinicians should consider training volume, as the intervention in the present study may not have been sufficient. Second, exoskeleton-assisted walking may not improve wheelchair function in individuals with chronic SCI. Specific wheelchair-based training is most likely the best intervention to improve wheelchair performance and abilities. Third, initial physical conditioning may also affect the response of upper limbs to exoskeleton-assisted walking. Individuals who are deconditioned are most likely to benefit the most from the intervention.

### Conclusions

The completion of an exoskeleton-assisted walking programme did not elicit significant and meaningful beneficial changes in upper limb muscle function or advanced functional wheelchair performance and abilities in long-term wheelchair users with chronic SCI. These findings confirm that the training volume (i.e., duration, frequency, intensity) may not be sufficient to improve upper limb muscle strength in this population. Nevertheless, no deleterious effects were found. Future research should focus on larger clinical trials, including a control group, to better determine the effects of the intervention, including whether it may contribute to maintaining upper limb strength and function. Researchers should consider a greater training volume, which will most likely require a longer intervention duration, to maximize potential effects. Moreover, researchers should consider the initial training status of their participants, as this will most likely influence the response to the intervention. Additional research is warranted with regard to relative muscle demand, as well as upper limb loading during overground walking with a robotic exoskeleton, especially in individuals who are experienced wheelchair users and who have acquired an efficient and safe walking technique with the exoskeleton.

## Trial registration

This trial was registered on clinicaltrials.gov (NCT03989752).
